# The Future of Aging Research

**DOI:** 10.1093/geroni/igaa057.2616

**Published:** 2020-12-16

**Authors:** Stephen Helfand, Jackson Taylor

## Abstract

We are at a particularly propitious time in the history of the biology of aging and the science of health and humanity. It is a time for us to “Honor the Past, Enrich the Future”. Not long ago the study of the biology of aging was exclusively one of description of what happened with age—how do organisms, including humans, change with age at the level of proteins, cells, tissues and physiology. The identification of major genetic factors that substantially increase life span in model organisms ushered in the advent of a highly exploratory period focusing on the molecular, cellular and genetics mechanisms of aging. The convergence of many different streams of basic and clinical research have brought us to today, where we stand on the cusp of new environmental, molecular genetic and pharmacological breakthroughs in the biology of aging that presage new interventions that promise a healthier lifespan. The presentations in this Presidential Symposium will be from four Early Career Investigators presenting their own pioneering research in some of the most important areas of research in the biology of aging.

